# Chronic Hepatitis B: Current Management and Future Directions

**DOI:** 10.3390/diseases13100311

**Published:** 2025-09-23

**Authors:** Hamza Ertugrul, Esra Ekiz, Sibel Islak Mutcali, Veysel Tahan, Ebubekir Daglilar

**Affiliations:** 1 Trinity Health of New England, St. Mary’s Hospital, 56 Franklin Street, Waterbury, CT 06706, USA; esra.ekiz@trinityhealthofne.org; 2 Vandalia Health, Charleston Area Medical Center (CAMC), 3100 MacCorkle Ave. SE, Charleston, WV 25304, USA; sibel.islak-mutcali@vandaliahealth.org (S.I.M.); veysel.tahan@vandaliahealth.org (V.T.); 3 Department of Internal Medicine, Division of Gastroenterology, Northeastern Ohio Medical University (NEOMED), 4209 OH-44, Rootstown, OH 44272, USA

**Keywords:** hepatitis B virus (HBV), covalently closed circular DNA (cccDNA), nucleot(s)ide analogs (NAs), pegylated interferon alpha (PegIFNα), functional cure, capsid assembly modulators (CAMs), HBsAg release inhibitors, farnesoid X receptor (FXR) agonists

## Abstract

Chronic hepatitis B virus (HBV) infection remains a major global health burden, affecting millions and contributing significantly to liver-related morbidity and mortality. While substantial progress has been made in elucidating the virology and natural history of HBV, the management of chronic hepatitis B (CHB) continues to present clinical challenges. The development of potent nucleos(t)ide analogs and pegylated interferon has improved viral suppression and delayed disease progression, yet a definitive cure remains elusive due to the persistence of covalently closed circular DNA (cccDNA). Recent research has focused on novel antiviral agents, immunomodulatory therapies, and combination strategies aimed at achieving a functional cure. This review summarizes current therapeutic approaches, recent advancements, and emerging directions in CHB management.

## 1. Introduction

Chronic hepatitis B (CHB) is a major contributor to cirrhosis, hepatocellular carcinoma (HCC), and liver-related deaths, with an estimated 780,000 fatalities each year [1]. Although effective vaccines and antiviral treatments exist, CHB continues to pose significant public health challenges, especially in areas where screening and sustained medical follow-up are limited.

The progression of CHB results from dynamics between viral replication, the host’s immune defenses, and the persistence of covalently closed circular DNA (cccDNA) within liver cells [2]. Clinical management is further complicated by variability in treatment response across different patient subgroups, risk of drug resistance with certain agents, and uncertainty regarding the optimal timing for treatment initiation and cessation.

Efforts to achieve sustained viral suppression and, ultimately, a functional cure are ongoing. However, the challenge remains to balance long-term viral control with minimizing adverse effects and maximizing patient adherence. As CHB poses unique risks in immunosuppressed, pregnant, and HDV co-infected populations—including viral reactivation, fulminant hepatitis, and vertical transmission, which require specialized treatment strategies [1,3]—these groups were excluded from our review to maintain focus on the general population. This review provides an updated synthesis of current management approaches for CHB, while highlighting emerging therapeutic strategies aimed at overcoming the limitations of existing treatments.

### 1.1. HBV Lifecycle

**Figure 1:**
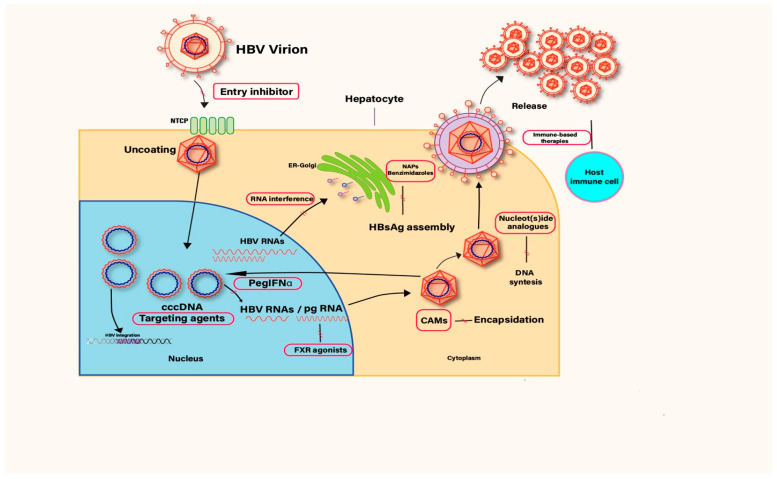
HBV lifecycle and associated treatment targets (CAM: Capsid assembly modulator, FXR: Farnesoid-X receptor. \\: Interfering with the following step/mechanism).

The hepatitis B virus (HBV) follows a coordinated life cycle involving complex interactions between viral structures and host cell mechanisms (Figure 1). Infection begins when HBV binds to the sodium taurocholate co-transporting polypeptide (NTCP) receptor on liver cells, enabling entry through endocytosis. Once inside, the viral nucleocapsid releases its relaxed circular DNA (rcDNA) into the cytoplasm, which is then transported to the nucleus. There, the rcDNA is repaired and converted into covalently closed circular DNA (cccDNA) [2], a stable mini-chromosome that serves as the blueprint for producing all viral RNAs, including pregenomic RNA (pgRNA) and messenger RNAs (mRNAs) encoding hepatitis B surface antigen (HBsAg), hepatitis B core antigen (HBcAg), and the polymerase enzyme. The pgRNA is packaged together with HBV polymerase in immature nucleocapsids, where reverse transcription converts it back into rcDNA. These nucleocapsids can either return to the nucleus to replenish the cccDNA pool or gain an envelope from surface antigens in the endoplasmic reticulum and Golgi, after which they are secreted as complete virions [4]. The durability of cccDNA within liver cell nuclei is the key reason HBV infections can persist for life, posing a major barrier to complete eradication.

### 1.2. Goals of Treatment

The primary objectives in managing CHB are to limit or reverse liver damage, prevent disease progression, and lower the risk of complications such as cirrhosis, liver failure, HCC, and death. These goals are achieved by reducing necro-inflammatory activity and slowing or reversing hepatic fibrosis. According to the 2020 Global Cancer Burden Report from the World Health Organization (WHO), liver cancer affected over 900,000 people globally and caused approximately 830,000 deaths, underscoring the seriousness of HBV-related disease. Notably, HCC caused by HBV tends to have poorer survival outcomes and higher recurrence rates after surgery compared with HCC from other causes [5]. This makes the pursuit of a “functional cure” an essential target. *Functional cure* refers to completing a finite treatment course, clearing hepatitis B surface antigen (HBsAg) with undetectable hepatitis B e antigen (HBeAg) and HBV DNA—regardless of anti-HBs status—and maintaining HBsAg negativity for at least 24 weeks after therapy ends [6]. While functional cure is the focus of newer strategies, understanding the different response types remains central to existing treatment approaches (Table 1).

### 1.3. Factors Predicting Response to Antiviral Treatment

A variety of patient-related and viral characteristics influence treatment outcomes in CHB. Currently, nucleos(t)ide analogs (NAs) and interferon (IFN) remain the mainstay therapeutic options. Across both approaches, lower baseline HBV DNA levels and elevated ALT are generally linked to better treatment responses, though important distinctions exist. For example, genotype A infections tend to have higher HBeAg and HBsAg seroconversion rates with PegIFNα compared to genotypes B, C, and D [7]. In HBeAg-positive patients on PegIFNα, a drop in HBsAg levels to below 1500 IU/mL by week 12 strongly predicts HBeAg loss and seroconversion, along with HBV DNA suppression, ALT normalization, and improved histology [8]. Conversely, HBsAg levels above 20,000 IU/mL or no decline at week 12 predict low likelihood of seroconversion [9]. Younger age, male sex, negative HBeAg at baseline, and coexisting hepatic steatosis are also favorable predictors for PegIFNα response [10,11].

In patients treated with NAs, certain factors—such as older age, presence of cirrhosis, and metabolic dysfunction-associated steatotic liver disease (MASLD)—may correlate with better outcomes. HBeAg quantity, rather than simple positive/negative status, can be informative; for instance, a fall in HBeAg by week 24 of tenofovir disoproxil fumarate (TDF) therapy is associated with later HBsAg loss after long-term treatment [12]. In lamivudine-treated patients, HBV DNA levels at six months are a key predictor of drug resistance [13]. For entecavir (ETV), favorable response factors include low baseline viral load, minimal fibrosis, negative HBeAg, and no prior IFN therapy [14,15].

It is important to note that the interplay of these factors can be complex, and individual patient responses to antiviral therapy can vary. Personalized treatment strategies based on individual patient characteristics and disease stage are essential for maximizing treatment success.

## 2. Current Treatment Options for CHB

The overarching aim of CHB therapy is to lower morbidity including cirrhosis, hepatic decompensation, liver failure, HCC, and enhance survival. This is primarily achieved by permanently suppressing HBV DNA to undetectable levels and achieving HBsAg loss. Additional goals of treatment include the reduction of necroinflammation and fibrosis, prevention of HBV transmission or reactivation, and improvement in life quality [1,16].

Currently, there are two main approaches: a fixed-duration course with PegIFNα or prolonged treatment with NAs. PegIFNα, ETV, and TDF are considered first-line therapies because they can halt liver injury progression and lower the risk of related complications. For patients with advanced disease, contraindications to interferon, or poor interferon tolerance, NAs are preferred [17]. NA therapy offers durable viral suppression, biochemical normalization, and histological improvement, as well as protection against decompensation, though HBsAg clearance remains uncommon. Table 2 summarizes approved antiviral therapies in adult population.

### 2.1. PegIFNα

PegIFNα works by blocking the transcription of pgRNA and subgenomic RNA derived from covalently closed circular DNA cccDNA. Approved by the U.S. FDA in 2005 for HBV treatment, PegIFNα is generally more effective than NAs at inducing both HBeAg and HBsAg seroconversion in HBeAg-positive patients. In contrast, its benefits are less pronounced in those who are HBeAg-negative. Among HBeAg-negative individuals with mild to severe liver disease, a 48-week course of PegIFNα can produce sustained virological response (SVR) in about one-quarter of cases, with 30–50% of those achieving SVR also clearing HBsAg. PegIFNα is not advised in patients with ALT levels exceeding five times the upper limit of normal, acute kidney injury, decompensated cirrhosis, or severe CHB flares [9].

### 2.2. Nucleot(s)ide Analogs

#### 2.2.1. Lamivudine (LAM)

A nucleoside analog reverse transcriptase inhibitor was the first oral antiviral therapy approved for CHB in 1998. After five years of therapy, about half of treated patients achieved HBeAg seroconversion. However, due to the high rate of resistance—occurring in roughly 70% of patients within five years—most current guidelines no longer recommend LAM as a first-line option [18].

#### 2.2.2. Telbivudine (LdT)

Approved in 2006, Telbivudine is another nucleoside analog closely related in structure to lamivudine, with a comparable resistance pattern. Because it shares cross-resistance with LAM, its use is generally limited to specific situations—most notably in pregnant women requiring HBV treatment—though it is less commonly used, as tenofovir has shown to be safe in pregnancy with high barrier properties [17].

#### 2.2.3. Entecavir (ETV)

Entecavir is a potent nucleoside analog that achieves rapid and sustained HBV DNA suppression, including in patients with HBV-related cirrhosis. In clinical studies, 96 weeks of therapy produced significantly greater improvements in patients with compensated cirrhosis compared with those who had decompensated disease [9,16].

#### 2.2.4. Adefovir Dipivoxil (ADV)

Adefovir dipivoxil can suppress HBV replication in both treatment-naïve patients and those with LAM-resistance. After one year, approximately 12% of HBeAg-positive patients achieve seroconversion, and more than half show histological improvement. However, its relatively high resistance rate and risk of kidney toxicity make it now rarely used as initial therapy, especially since more potent and safer options like ETV and TDF are available [19].

#### 2.2.5. Tenofovir Disoproxil Fumarate (TDF)

Tenofovir disoproxil fumarate is a potent nucleotide analog with strong antiviral efficacy. In comparative studies, TDF achieved higher viral suppression rates than adefovir—93% vs. 63% in HBeAg-negative patients and 76% vs. 13% in HBeAg-positive patients. Long-term data show that suppression is sustained in nearly all patients: 99% of those HBeAg-positive and 100% of those HBeAg-negative after four years of therapy. Notably, five years of TDF treatment has been associated with reversal of cirrhosis in about three-quarters of patients who had cirrhosis at baseline [20].

### 2.3. New Antivirals in Use

#### 2.3.1. Tenofovir Alafenamide (TAF)

Tenofovir alafenamide is a prodrug of TDF. With high intracellular concentration, it is found to be noninferior to TDF in both HBeAg-negative and -positive patients in achieving and sustaining virological response (i.e., HBV DNA < 29 IU/mL) and provided higher ALT normalization rates, according to American Association for the Study of Liver Diseases (AASLD) criteria, with a better renal side effect profile [17].

#### 2.3.2. Besifovir

Besifovir is a new oral nucleotide analog, which inhibits a reverse transcriptase enzyme of HBV, inhibiting new viral DNA production. In a retrospective study from South Korea—where the molecule is approved for treatment for CHB—it has shown to be noninferior to TAF in terms of viral response in treatment-naïve patients with CHB, where viral response is defined as undetectable serum HBV DNA [21]. Another multicenter trial, where Besifovir and ETV were compared, had shown that Besifovir had similar virological and biochemical response profile with ETV and was well-tolerated [22]. With its comparable renal safety to TAF, it is a promising drug for both treatment-naïve and LAM-resistant CHB patients; however, it has not yet been approved by Food and Drug Administration (FDA), due to a lack of multicenter phase III clinical trials to demonstrate its efficacy, safety, and long-term outcomes. Another obstacle is that Besifovir has mainly been studied in Asian populations, making the existing studies not generalizable.

## 3. Emerging Therapies: What Is on the Horizon?

Long-term viral suppression has not eradicated the virus, and there is need to develop new strategies achieving more than viral suppression.

Functional cure, as previously defined above, requires undetectable serum DNA, but ccc-DNA persists [23]. Lowering liver cccDNA levels, inactivating cccDNA-directed transcription to prevent viral replication, and inducing a remission of liver disease are the end-points of functional cure; however, “lowering” cccDNA levels are not enough to eradicate HCC risk [24].

Complete cure means the physical elimination of cccDNA, on top of a functional cure. Current antiviral treatment strategies do not provide a complete cure.

Clinical trials have been evaluating new classes of agents to target different steps in the HBV lifecycle. Table 3 summarizes the existing and emerging therapeutic agents targeting different stages of the HBV lifecycle with the current study phases for each group of molecules, if applicable.

### 3.1. Novel Direct-Acting-Antivirals (DAAs)

#### 3.1.1. Targeting cccDNA

HBV cccDNA is protected within the nucleus of hepatocytes and drives continuous transcription of viral proteins; in this way, treatments targeting cccDNA have gained much attraction.

1) **Nitazoxanide:** As an antiparasitic drug, nitazoxanide has inhibited degradation of a structural maintenance of chromosomes complex, which blocks HBV RNA transcription. In one pilot study, after 48 weeks, HBV DNA became undetectable in almost 90% of the treatment-naïve participants with CHB [25,26]. It requires further large-scale studies for its approval for antiviral indication.
2) **DNA cleavage enzymes:** Zinc-finger-like nucleases (ZFNs), transcription activator-like effector nucleases (TALENs), and clustered regularly interspaced short palindromic repeats (CRISPR)-associated system 9 (Cas9) proteins are among the DNA cleavage enzymes targeting cccDNA [9]. Currently in preclinical phases, studies have shown the CRISPR-Cas9 system being effective in HBV quasi-species [27]. However, safe and efficient delivery to infected hepatocytes in humans—without inducing host immune reactions to the gene-editing components and preventing off-target effects and genotoxicity of such molecules in general—remains to be an obstacle to translational studies [28].
3) **APOBEC3 (A3) enzymes:** Apolipoprotein B mRNA editing catalytic polypeptide-like (APOBEC) deaminases can degrade cccDNA without damaging hepatocytes [29]. Certain interferons (alpha, beta, lambda) can stimulate APOBEC3 activity, promoting cccDNA destruction [30]. Off-target mutagenesis induced by APOBEC3 enzymes in the host genome, along with variable expression of these enzymes—which can be suppressed by factors such as hypoxia-inducible factor 1 alpha (HIF1α), upregulated in chronic liver disease and impairing APOBEC3-mediated antiviral effects—are the main barriers to therapeutic use [31,32].

#### 3.1.2. Targeting Anti-Apoptosis Proteins

HBV-infected cells often survive longer than normal cells due to proteins that block programmed cell death. One such group, cellular inhibitors of apoptosis proteins (cIAPs), interferes with the tumor necrosis factor (TNF)-mediated killing of infected cells. Drugs known as SMAC mimetics (second mitochondrial-derived activators of caspase) imitate the action of natural cIAP inhibitors and can help clear infected cells [33]. Birinapant, a SMAC-mimetic, in early trials, reduced HBV DNA and HBsAg levels and enhanced the antiviral effect of ETV [34]; however, these findings have not yet been translated into robust human clinical trial data.

#### 3.1.3. Capsid Assembly Modulators

CAMs disrupt the assembly of HBV’s protective capsid, preventing the formation of viable viral particles. Two main classes exist as follows:

*Core protein allosteric modulator-I or heteroaryldihydropyrimidines (CpAM-I or HAPs):* Cause aberrant, nonfunctional core proteins.
*CpAM-II or phenylpropenamides-PPAs and sulfamolybenzamides-SBAs):* Trigger formation of capsids without viral DNA [35,36,37].

*Studies with representatives of HAP family:*

1) **RO7049389:** RO7049389 is an oral molecule studied in a multi-center randomized controlled (RCT) phase I study, where treatment with this molecule led to a decrease in HBV DNA and RNA, but there was no change in HBsAg levels. Post-treatment observation showed viral rebound to pretreatment levels [38,39].
2) **GLS4:** In combination with ritonavir, it modestly lowered HBV DNA, HBsAg, and HBeAg levels, though the effect was less pronounced when compared to ETV alone. Nevertheless, relapse occurred after stopping therapy [40,41].
3) **Bay41–4109:** This is a molecule tested only in preclinical studies, and shown to reduce HBV replication and intracellular HBV RNA, HBV antigenemia, and cccDNA formation [42].

*Studies with representatives of SBA family:*

1) **NVR 3–778:** This molecule, in combination with Peg-IFNα, is shown in a phase 1b trial to induce greater suppression in viremia and HBV RNA; however, HBsAg and HBeAg did not change significantly within 28 days of treatment [43].
2) **Bersacapavir (JNJ6379):** Bersacapavir is a CAM that induced defective capsid formation, reducing cccDNA [44]. In a double-blind study, it resulted in a decline in viremia; however, viral loads returned to baseline after stopping therapy [45]. It is now being tested in combination with NAs and siRNA agents.
3) **Vebicorvir (ABI-H0731):** Vebicorvir monotherapy showed strong initial viral suppression but relapse after discontinuation; combination regimens are under investigation. In a 24-week phase II trial, a combination of the novel core inhibitor vebicorvir with ETV in treatment-naïve patients with HBeAg positive CHB infection demonstrated greater HBV DNA reduction and increased ALT normalization rates over ETV alone. However, most of the patient population were of Asian descent, and findings cannot be generalized to a global population [46].

#### 3.1.4. RNA Interference

RNA interference (RNAi) drugs aim to reduce HBV protein production by breaking down viral messenger RNA (mRNA) or blocking its translation. This limits the production of key antigens such as HBsAg and HBeAg, reducing viral replication and release [47].

1) **ARC-520:** In a phase II clinical trial, ARC-520 reduced HBsAg in chimpanzees and patients who were HBeAg-positive, but the effect was not as much pronounced in HBeAg-negative ones [48]. Due to dose-dependent and delayed hypersensitivity reactions observed in animal models caused by EX1 excipient of ARC-520, its clinical development is discontinued [49].
2) **JNJ-3989:** In a phase II study, JNJ-3989 is shown to reduce HBsAg, and a sustained HBsAg reduction was observed in more than 50% of the patients after cessation of treatment. When combined with TDF or ETV, it induced sustained HBV-RNA and HBeAg reduction; however, it has not achieved HBsAg sero-clearance off-treatment [50].
3) **Imdusiran (AB-729):** Imdusiran is a small interfering RNA (si-RNA) therapeutic that resulted in HBsAg decline without rebound rise post-treatment. When given as a single dose to HBeAg-negative patients with low viremia, it also led to a significant drop in HBsAg and undetectable HBV RNA levels in all patients up to 36 weeks. It was active against NA- and CAM-resistant HBV isolates, and combination with standard-of-care agents was additive. Current data do not meet regulatory requirements for market approval yet [51,52].
4) **Bepirovirsen (GSK3228836):** It is an antisense oligonucleotide molecule that targets HBV mRNAs and acts to decrease viral protein levels. In a phase 2b trial, Bepirovirsen was given to both NA-naïve patients and individuals who were already on NA treatment, resulting in sustained HBsAg and HBV DNA loss in a small proportion of participants (10%) [53]. Bepirovirsen received a US-FDA Fast Track designation as of 2024 [54].

#### 3.1.5. Host-Targeted Therapies

1) **Viral Entry Inhibitors:** Entry inhibitors act in various ways, mainly by interfering with peptides involved in HBV entry; however, they do not affect cccDNA. Thus, their utility would be in combination regimes with molecules that target cccDNA formation or degradation.
  - **Bulevirtide (Myrcludex):** Sodium-taurocholate co-transporting polypeptide (NTCP) was identified as a host entry factor for HBV. Bulevirtide inhibits NTCP receptor binding, blocking HBV entry [55,56]. It showed HBV DNA decline in HBeAg-negative CHB patients but minimal effect on HBsAg [57]; hence, studies by and large focus on hepatitis D virus (HDV) co-infection, and it is now approved for chronic HDV infection in Europe [58].
  - **Monoclonal antibodies:** Neutralizing antibodies can inhibit HBV entry and target viral envelope antigens recognizing various HBsAg epitopes, stimulating adaptive immunity. Current studies are in phase I or II [59].
  - **Cyclosporine:** CysA derivatives without immunosuppressant activity can prevent HBV attachment to NTCP [60]; however, they have not advanced to human trials or regulatory review yet.
2) **HBsAg release inhibitors:**
  - **Nucleic acid polymers (NAPs):** HBsAg both forms the surface of HBV virions and allows entry to hepatocytes via NTCP receptor. Blocking HBsAg release would prevent release of further enveloped viruses. DNA or RNA-based NAPs, which block release of HBsAg, are now being studied in trials for mono or combination therapy [61]. A phase 2 pilot study combined a NAP with either PEG-IFNα or TDF in HBeAg-negative treatment-naïve CHB patients, which resulted in HBsAg seroconversion in all patients [62]. Larger multicenter studies needed for the characterization of long-term safety—including possible HBsAg accumulation in hepatocytes causing ALT-flares—and to confirm HBsAg loss after treatment cessation [63].
  - **Benzimidazoles: BM601**, a secretion inhibitor, inhibits transport of the HBV surface protein to the Golgi apparatus, lowering HBsAg and virion release without affecting HBeAg [64]; however, this effect has not been validated in animal or human models yet.
3) **Farnesoid X receptor (FXR) agonists:** Bile acid nuclear receptor FXR binds to cccDNA and enhances HBV transcription. **Vonafexor** is a FXR-agonist, which, in phase II trials with PegIFNα and entecavir, showed strong HBV DNA and HBsAg reductions in HBeAg-positive patients, with smaller effects in HBeAg-negative individuals [65]. The risk of hepatotoxicity via mitochondrial dysfunction, hepatocyte apoptosis—especially at higher doses or with chronic administration—and potential metabolic side effects—due to their central role in bile acid, lipid, and glucose metabolism—remain to be an obstacle for FXR agonists [66].
4) **Cyclophilin inhibitors:** Cyclophilins are host proteins that catalyze cis- to trans- conformation in protein folding and participate in cellular signaling and immunomodulation. There is substantial data that shows cyclophilin involvement in HIV and HCV infection [67]. Cyclophilins are also implicated in the HBV lifecycle. One molecule in this group, ***CRV341***, is being studied in MASLD and HCC. In HBV transgenic mice, CRV431 reduced intrahepatic HBV DNA and moderately decreased serum HBsAg, with additive effects when combined with tenofovir analogs and no observed toxicity in these models [68].

### 3.2. Immune-Based Therapies

#### 3.2.1. Activation of the Innate Immune System

1) **Toll-like receptor (TLR) agonist:** TLRs are expressed on macrophages and dendritic cells and recognize molecules from microorganisms. TLR-7 and 8 induce expression of genes involved in antiviral cytokine release. TLR-7 agonists studied are as follows: ***RO702053, JNJ-4964, and GS-9620 (Vesatolimod)***. *Vesatolimod* has shown to increase HBV-specific T cells but there was no significant drop in HBsAg levels [69]. TLR-8 agonist ***Selgantolimob*** was studied in HBeAg + CHB patients as a monotherapy, but the effect on HBsAg decline or HBeAg seroconversion was not satisfactory; however, when combined with TAF, there was a more pronounced reduction in HBsAg [70]. Combination regimens are being investigated, with the rationale that reducing antigen load with direct-acting antivirals may enhance the efficacy of immunomodulatory agents; however, sustained HBsAg loss has not been achieved yet to bring these molecules close to active clinical use.
2) **Retinoic acid inducible gene-1 (RIG-1) agonist:** RIG-1 is an intracytoplasmic dsRNA sensor, which plays a role in immune response to CHB. After activation, it induces cytokine production via intracellular pathways, especially IFN-l production, which is known to inhibit HBV replication directly and to activate innate and adaptive immunity [71]. A RIG-1 agonist, ***Inarigivir***, has been studied in HBeAg-positive and -negative patients, as monotherapy, followed by switching to TDF. The study showed a reduction in HBV DNA and RNA; however, it was found to have significant hepatotoxic side effects. Therefore, the study was terminated [47].
3) **Programmed death-1 (PD1) inhibition:** The immune checkpoint receptor (ICR) blockade has opened a new era in cancer treatment. As immune response to HBV plays a major role in the development of CHB, the same pathway gained attraction in recent investigations. One of them showed programmed death-1 (PD1) being correlated with viremia and HBeAg and decreased with HBeAg seroconversion. In this context, PD1 inhibitor ***nivolumab*** was studied; however, it has only shown minimal decline in HBsAg [72].

#### 3.2.2. Activation of the Adaptive Immune System

1) **The IFN system:** Despite PegIFNα being a second-line agent in CHB treatment due to its side effect profile, it is the only approved drug with finite duration. PegIFNα is now being studied in combination with other antiviral agents. IFN-λ has similarities with IFN-a but has fewer side effects due to its more restricted expression in epithelial and immune cells. Moreover, it was evaluated in a study where it showed earlier decline in viral load and similar HBeAg seroconversion when compared to PegIFNα [73]. However, based on post-treatment seroconversion, virologic suppression, and biochemical response rates, PegIFNα2a demonstrated greater overall efficacy. Nonetheless, findings suggest a potential role for IFN-λ, especially when combined with NAs, in supporting immune-mediated control of HBV, which may lead to the suppression of cccDNA activity [74].
2) **Therapeutic vaccination:** This approach seeks to retrain the immune system to recognize and attack HBV. Among these are GS-4774, ABX-203 (HeberNasvac), BRII-179, TG 1050, VTP-300, and TherVacB. Despite inducing strong immune response in unaffected individuals, HBV vaccines failed to show a benefit in HBV-infected patients until now.
  - **GS-4774,** containing HBsAg and HBcAg, did not show superiority in HBsAg decline when compared to patients treated with NAs-only [75].
  - **ABX-203 (HeberNasvac),** containing HBsAg and HBcAg, resulted in equally suppressed HBV DNA levels when compared with PegIFNα alone but did not result in loss of HBsAg [76].
  - **BRII-179,** containing HBsAg, induced cellular and humoral immune response but did not result in a significant change in HBsAg levels [77].
  - **TG1050,** an adenovirus-based vaccine, expressing HBsAg, HBcAg, and HBV polymerase, resulted in minor decrease in HBsAg [78].
  - **VTP300,** an immunotherapeutic vaccine, has demonstrated to lower HBsAg levels both as monotherapy and in combination with Nivolumab or Imdusiran, with the combination resulting in sustained HBsAg declines [79].
  - **TherVacB,** a modified vaccinia virus Ankara (MVA)-vector boosted heterologous vaccine, elicited HBV-specific antibody and T cell responses in wild type and HBV-carrier mice [80]. The first clinical trial with TherVacB started in 2024.

## 4. Conclusions

The management of CHB has significantly evolved over recent years, with NAs and PegIFNα remaining the cornerstone of current treatment strategies, aimed at long-term viral suppression and prevention of disease progression. However, a complete virological cure remains elusive for most patients. Emerging therapies targeting novel aspects of the HBV life cycle hold promise to achieve functional cure by inducing sustained HBsAg loss. Nevertheless, there is no evidence of clinically meaningful differences in efficacy among HBV genotypes for the investigational agents. As these innovative therapies advance through clinical development, a paradigm shift towards finite, curative regimens are anticipated. Continued research into HBV pathogenesis and host immune response, along with combination approaches integrating both antiviral and immune-based therapies, will be pivotal in transforming the landscape of CHB management in the coming years.

## Figures and Tables

**Table 1:** Definition and types of response (*adapted from [7]*).

Response Type	Definition
**Biochemical response**	ALT returns to normal range
**Serologic response**	Loss of HBsAg with anti-HBs development, or loss of HBeAg with anti-HBe development in HBeAg-positive patients
**Virologic response to Peg-IFN**	HBV DNA <2000 IU/mL; if maintained ≥12 months after treatment completion, termed sustained virologic response
**Virologic response to nucleos(t)ide analogues (NAs)**	Undetectable HBV DNA
**Partial virologic response (to NAs)**	≥1 log_10_ IU/mL HBV DNA decline, but still detectable after ~24 weeks of NA therapy
**Complete response**	Sustained virologic suppression with HBsAg seroconversion
**Sustained off-treatment response**	No relapse observed during follow-up after therapy is stopped
**Histologic response**	≥2-point reduction in necroinflammatory score without fibrosis worsening, or ≥1 stage improvement in fibrosis by METAVIR
**Primary non-response (to NAs)**	HBV DNA decline <1 log_10_ IU/mL after 12 weeks of therapy
**Virologic breakthrough**	≥1 log_10_ IU/mL increase in HBV DNA from lowest level while on therapy
**Viral relapse**	HBV DNA >2000 IU/mL after stopping therapy in a patient who had prior virologic suppression
**Clinical relapse**	Viral relapse accompanied by ALT elevation > 2 × ULN

**Table 2:** Brief summary of approved antiviral therapies in adults (*adapted from [17]*).

Drug	Pregnancy Category	Major Side Effects	Suggested Monitoring
Pegylated IFN-α2a	C	Flu-like illness, mood/psychiatric changes, blood count suppression, autoimmune phenomena	CBC * and TSH ** every 3 months; clinical surveillance for neuropsychiatric, autoimmune, or infectious complications
Lamivudine	C	Risk of pancreatitis, lactic acidosis	Amylase if symptomatic; lactate when clinically indicated
Telbivudine	B	CK *** elevation, muscle toxicity, neuropathy; lactic acidosis	CK if symptoms develop; lactate if clinically indicated
Entecavir	C	Lactic acidosis (rare)	Lactate if clinically indicated
Adefovir	C	Nephrotoxicity (acute renal failure, Fanconi-like syndrome), tubular dysfunction, lactic acidosis	Baseline CrCl ****; periodic monitoring of renal function, phosphorus, urine glucose/protein (especially in high-risk patients); bone mineral density (BMD) if fracture/osteoporosis risk; lactate if clinical concern
Tenofovir	B	Renal injury (nephropathy, Fanconi syndrome), bone loss/osteomalacia, lactic acidosis	Baseline and periodic CrCl; phosphorus and urine markers yearly if risk present; consider baseline BMD in high-risk groups; lactate if clinically indicated

* CBC: Complete blood count, ** TSH: Thyroid stimulating hormone, *** CK: Creatine kinase, **** CrCl: Creatine clearance.

**Table 3:** Summary of existing and emerging therapeutic agents for CHB with trial phases, end points and estimated costs.

Agent/Class	Representative	Trial/Registry	Phase	Primary Endpoints/Outcome Notes	Cost Estimate *	Readiness
**Interferons**	PegIFNα2a (Pegasys ^®^)	NCT00487747	Approved	HBeAg seroconversion, HBsAg decline, ALT normalization	~$1336/dose (US list price)	Market (Approved)
**NAs (Nucleos(t)ide analogs)**	Lamivudine	Historic pivotal	Approved	HBV DNA suppression < LOD ** at Wk *** 48; effective but resistance issues	~$16/day (generic US)	Market (Approved)
	Telbivudine	Historic trials	Approved	HBV DNA suppression; HBeAg seroconversion; resistance limits use	~$20–30/day	Market (Approved; supplanted)
	Entecavir	Pivotal	Approved	HBV DNA < LOD at Wk 48; high potency, low resistance	~$2–3/day (generic)	Market (Approved)
	Adefovir dipivoxil	Historic	Approved	HBV DNA suppression at Wk 48; supplanted by newer agents due to potency/safety	~$35–40/day	Market (Older)
	Tenofovir disoproxil fumarate (TDF)	Pivotal	Approved	HBV DNA < 29 IU/mL at Wk 48 (noninferiority design); high potency, long term safety concerns	~$3–4/day (generic)	Market (Approved)
	Tenofovir alafenamide (TAF, Vemlidy ^®^)	Phase 3 pivotal	Approved	HBV DNA < 29 IU/mL at Wk 48, ALT normalization; safer kidney/bone profile	~$19–20/day	Market (Approved; generics emerging)
	Besifovir	NCT01937806 (Phase 3 registry)	Phase III/Korea approval	Virological response (HBV DNA < threshold at Wk 48); non-inferior to TDF	Projected ~$15–20/day	Near (regional approval in Korea, not FDA/EU ****)
**Novel DAAs**	Nitazoxanide	Small Phase 2	Phase II/pilot	HBV DNA decline, HBsAg change; weak pilot data shows antiviral signal	<$10/day (generic antiparasitic)	Far (investigational for HBV)
	DNA cleavage (CRISPR, TALEN, etc.)	Preclinical	Preclinical	cccDNA cleavage; antigen reduction. Promising, but delivery issues	N/A	Far
	APOBEC3-based approaches	Preclinical	Preclinical	cccDNA deamination; promising in vitro/in vivo	N/A	Far
**Capsid assembly modulators (CAMs)**	ABI-H0731 (Vebicorvir), JNJ-56136379 (Bersacapavir), RO7049389, GLS4, BAY41-4109, NVR-3-778	NCT04820686 (ABI combos), NCT04667104 (JNJ combos)	Phase I–IIb/some combo studies	HBsAg/HBV DNA reduction at Wk 24/48; relapse issues	Projected ~$50–100/day	Medium (Phase II; combo trials progressing)
**Apoptosis/cIAP inhibitors**	Birinapant, ABT-869, etc.	Early exploratory	Phase I	Safety; exploratory antiviral endpoints; enhances efficacy of ETV	Projected ~$50–200/day	Far (early)
**RNA interference (RNAi)**	JNJ-3989 (JNJ-73763989), AB-729 (Imdusiran)	NCT04980482	Phase II	HBsAg mean log decline at Wk 12–24, safety. Strong HBsAg reduction when added to standard of care	Projected siRNA pricing ~$500–1000+/month	Medium (promising durability signals in Phase II)
	GSK3228836 (Bepirovirsen)	B-Clear Ph2b (NCT04449029)	Phase II → III initiated	HBsAg reduction/loss; FDA fast track	Projected ~$1000–2000+/month	Medium → Near (Phase III in progress)
**Viral entry inhibitors**	Bulevirtide (Hepcludex ^®^)	EMA ***** HDV trials	Approved (HDV)/HBV investigational	HDV RNA decline, ALT normalization	~$46k/year list price	Near (EU approval in 2020; FDA pending)
**Monoclonal antibodies**	Anti-HBs mAbs	Multiple Phase I-II	Phase I–II	Safety; HBsAg neutralization/decline. Studies in combo for finite therapy	Projected biologic pricing: Hundreds–thousands per dose	Far → Medium
**Cyclophilin inhibitors**	Cyclosporine	Early repurposing studies	Early repurposing studies	Safety, viral load endpoints in small cohorts; mixed data	Generic cyclosporine inexpensive, not priced as antiviral.	Far
	CRV431	Phase I registry	Phase I	Safety; viral load endpoints; pharmacokinetics	Projected pricing ~$50-200/day	Far
**HBsAg release inhibitors (NAPs)**	REP-2139	Phase II combo	Phase II	HBsAg decline/loss; anti-HBs seroconversion. Promising combo with PegIFN2α	Projected ~$500–1000+/month (complex biologic/nucleic acid therapy)	Medium (small promising trials, safety/logistics considered)
**FXR agonists**	Vonafexor	Phase II	Phase II	HBV DNA/HBsAg change; more effective in HBeAg+	Projected small molecule pricing~$300–600/month	Medium
**Innate immune activators**	TLR agonists (GS-9620-Vesatolimod, Selgantolimod, RO702053, JNJ4964)	Various NCTs	Phase I–II	Safety; immune activation; HBsAg/HBV DNA changes. Long term sustained efficacy is not proven	Projected ~$200–500/dose	Medium → Far (modest single-agent efficacy)
	RIG-I agonist (Inarigivir)	Early NCTs	Early/development holds	Safety; HBsAg/HBV DNA endpoints; hepatotoxicity issues	Projected small-molecule pricing ~$50–200/day	Far/uncertain
**Adaptive immunity activators**	PD-1 inhibitor (Nivolumab)	Small NCTs	Phase I	Safety (hepatic flares), immune response markers	Oncology biologic pricing $10k–20k/dose, HBV use experimental	Far/experimental
	IFN-λ (pegylated)	Phase II	Phase II	HBsAg decline/loss, ALT normalization; better tolerated than PegIFNα2a	Projected PegIFN pricing ~$1000–3000/course	Medium
**Therapeutic vaccines**	GS-4774, ABX-203 (HeberNasvac), BRII-179, TG1050, VTP-300, TherVacB	Multiple/Representative NCTs per vaccine	Phase I–II (some regional Phase II/III for HeberNasvac)	Safety; immune biomarker responses; HBsAg changes/conversion; modest single-agent efficacy, combination approaches favored	Projected vaccine program pricing $50–1000+/course	Far → Medium

* Cost estimates are obtained from GoodRx, comparator pricing from the same group of agents that are already in market for other indications, ** LOD: Level of detection, *** Wk: Week, **** EU (European Union), ***** EMA (European Medicines Agency).
